# The Preservation of Functional Reserve: A Control Systems Framework for Human Aging

**DOI:** 10.3390/life16091457

**Published:** 2026-08-31

**Authors:** Robert T. O’Leary

**Affiliations:** Orlando College of Osteopathic Medicine, Orlando, FL 34787, USA; dr.oleary@gmail.com

**Keywords:** functional reserve, homeostenosis, mitochondrial–epigenetic axis, control systems, physiologic resilience, longevity medicine

## Abstract

The dominant clinical manifestation of aging is not mortality but the progressive depletion of functional reserve—the surplus physiological capacity separating independent function from disability. Existing geroscience frameworks describe molecular aging in detail yet give the point-of-care clinician little basis for prioritizing targets. We reframe functional aging as a control systems problem in which aging is modeled as progressive instability within a coupled, bidirectional mitochondrial–epigenetic axis. That axis is called central in two limited senses only: network connectivity and therapeutic tractability; no causal primacy is claimed over mTOR signaling, inflammaging, senescence, proteostasis failure, telomere attrition, or stem cell exhaustion. Candidate capacities were scored against three conjunctive criteria—independence, necessity, and modifiability. Five satisfy them at the thresholds stated, and sensitivity analysis reports which threshold changes would instead yield four or seven, so the count follows from a stated procedure rather than standing as a natural constant. Interventions are mapped onto these capacities in a separate layer. The result is a hierarchically ordered, clinically actionable, falsifiable architecture in which functional reserve is the primary therapeutic target and lifespan a downstream consequence—and whose primary test, deletion of any single capacity from longitudinal models, is evaluable in existing cohorts without new data collection.

## 1. Introduction

The twentieth century’s signature medical achievement—the dramatic extension of human lifespan—has produced an unanticipated burden: large populations living longer without a corresponding compression of morbidity. The central clinical problem of aging is not death; it is the progressive, systematic erosion of the capacity to function—to perform activities of daily living, maintain independent mobility, preserve executive cognition, and sustain physiological resilience under stress.

Several influential frameworks have advanced the mechanistic understanding of aging. The hallmarks of aging [1,2] catalog the cellular and molecular processes; information-theoretic models demonstrate that loss of epigenetic information is itself a cause of aging [3]; and mitochondrial theories emphasize declining bioenergetic capacity and impaired quality control. Each is indispensable, yet none translates the complexity of molecular aging into a prioritized, hierarchically ordered set of targets usable at the point of care.

We propose that functional aging is more productively framed as a control systems problem. The clinically decisive question is not how to prevent every molecular defect, but how to maintain stability within the control systems that preserve organized function across decades. This reframing inverts the clinical orientation—from “what goes wrong during aging?” to “what must be preserved for function to endure?”—and, as developed below, makes functional reserve the explicit organizing outcome.

## 2. Functional Reserve: The Central Construct

**Definition** **1.** *Functional reserve is the excess physiological capacity available beyond that required for basic independent function. It is the buffer that allows an organism to absorb acute stressors—infection, injury, surgery, metabolic challenge—and return to baseline without crossing into dependency*.

This construct is not novel in isolation, and the framework does not claim it as original. Geriatric medicine already names physiologic reserve, describes its progressive narrowing as homeostenosis—the age-related contraction of the homeostatic operating range—and operationalizes its depletion as frailty [4,5]. The contribution here is architectural: to make reserve the explicit terminal outcome of the model and to specify the control systems that generate and defend it.

Relation to the complexity loss framework. The proposal that aging is characterized by a progressive loss of physiological complexity is well established, and any account of declining adaptive capacity must situate itself against it [6,7,8]. That framework describes the dynamical signature of regulation: healthy output fluctuates with structure correlated across multiple temporal scales, and that structure simplifies with age and disease, narrowing the range of adaptive responses available to a stressor. The present framework describes a different quantity at a different level of description—the magnitude of the margin between attainable capacity and the demand at which function fails. The two are complementary rather than competing. A controller with fewer available modes exerts less authority over the same plant, so loss of complexity is plausibly one mechanism by which reserve narrows; conversely, a wide margin permits the exploratory, multi-scale regulation that complexity metrics detect. The distinction that follows is practical. Complexity is read from the fluctuation structure of a single recording. Reserve is read only by loading the system toward its limit, and its trajectory only by loading it repeatedly over time.

One claim advanced here is not contained in that prior framework: that the margin is use-dependent and hormetic—built by demand, shed in its absence, and degraded by excess as well as by insufficiency.

Measurable proxies. Reserve is already instrumented by markers validated in the prospective mortality literature: VO2 max [9], grip strength, gait speed [10], cognitive reserve, metabolic flexibility, and heart rate variability. The framework therefore inherits the predictive validity of these established outputs rather than proposing new ones.

The reserve view of aging. Aging is the progressive depletion of reserve toward the disability threshold. Crucially, interventions act not by abolishing molecular damage but by widening or refilling reserve—raising the ceiling, slowing the rate of drawdown, or restoring lost capacity. This reframes the therapeutic objective in terms a clinician can measure and a patient can experience.

## 3. Aging as Control Failure

A control system maintains a regulated variable within bounds despite disturbance, using sensors, a controller with defined gain, and actuators. Youthful physiology approximates a robust controller: wide reserve, intact feedback, and rapid correction. Aging is the degradation of that controller—and homeostenosis is its signature. The framework identifies four canonical failure modes, each mapping to recognizable aging biology (Table 1):

The clinical inversion follows directly: the physician’s task is not to repair every cellular defect but to keep the controllers stable and reserve above the disability threshold. The remainder of this paper specifies which controllers, in what hierarchical order, and how they are modified.

On the register of “control systems.” We use control systems vocabulary as an organizing formalism at the level of failure modes, and it is important to be explicit about what that does and does not supply. This paper does not present a formal systems model: it identifies no state vector, specifies no transfer functions, estimates no feedback gains or time constants, and performs no stability analysis. A reader arriving from control engineering will find the terms used in their qualitative sense—regulated variable, sensor, controller, actuator, setpoint—rather than their mathematical one.

The claim is that this vocabulary organizes aging biology more usefully for a clinician than a descriptive catalog does, because it directs attention to which loop has degraded rather than to which molecule is abnormal. That claim stands or falls on clinical utility, not on mathematical completeness. Formalizing the framework would require, at minimum, longitudinal multi-determinant measurement in the same individuals at sufficient density to estimate loop gain and recovery time constants after defined perturbation—measurements that do not currently exist at scale. We identify this as the principal direction in which the framework would become a model rather than an architecture, and Limitation 5 states the instrument gap that presently prevents it.

## 4. The Central Regulatory Axis: Mitochondrial–Epigenetic Coupling

We hypothesize that functional aging emerges from progressive instability within a tightly coupled, bidirectional mitochondrial–epigenetic axis:

Mitochondrial dysfunction → ↓ NAD+ → ↓ sirtuin activity → epigenetic drift → impaired mitochondrial biogenesis and mitophagy → mitochondrial decay.

Declining oxidative phosphorylation reduces NAD+ availability, impairing sirtuin deacylase activity [11,12]. Reduced sirtuin activity accelerates epigenetic drift and loss of transcriptional fidelity [3,13,14]. Epigenetic disorganization, in turn, impairs mitochondrial biogenesis and quality control [15]—closing and amplifying the loop.

A thermodynamically disciplined statement. Living cells are open, dissipative systems: they sustain their highly ordered state only by continuously expending free energy on active maintenance and repair. Mitochondria supply that free energy as ATP. When supply falls, the rate of active correction drops below the rate of spontaneous disordering, and organized function erodes. We deliberately avoid framing this as a mystical “negentropy engine”; the claim is the conventional one that maintenance is energetically costly and the mitochondrion is its principal funder.

What “central” means here—and what it does not. We use central in two limited senses, and in neither does it mean causal primacy. The first is network connectivity: the axis carries direct, bidirectional mechanistic edges to a larger number of the processes cataloged as hallmarks of aging than those processes carry to one another, which makes it an efficient point of organization for a clinician who must prioritize. The second is therapeutic tractability: the axis is reachable by interventions of established human safety—physical activity, circadian and sleep regulation, energy balance, thermal and hypoxic conditioning—whereas several rival organizing candidates are at present addressable in humans only experimentally.

We explicitly do not claim that the axis initiates aging, nor that it sits causally upstream of mTOR signaling, chronic inflammation, cellular senescence, proteostasis failure, telomere attrition, or stem cell exhaustion. Limitation 3 states the contrary possibility and it remains open. A framework organized around a hub selected for connectivity and tractability makes a claim about where intervention is most efficient, not about first causes. It can be wrong about causation and still be right about where to act—and, importantly, it can be shown to be wrong about tractability, which is the claim Section 11 tests (Table 2).

**Table 2:** Rival organizing candidates and their relation to the mitochondrial–epigenetic axis.

Candidate Hub	Relation to the Axis	Why Not Selected as Hub
mTOR signaling	Upstream nutrient sensor; gates autophagy/mitophagy (Det. III) and mitochondrial biogenesis (Det. I)	Strong connectivity, low present tractability: chronic mTOR inhibition is not an established intervention in healthy humans
Chronic inflammation (inflammaging)	Read here as an integrated output of uncompensated failure across I–V	High connectivity but principally an output; intervening on it does not restore the capacities generating it
Immune aging (immunosenescence)	Thymic involution and impaired clearance are conditioned by bioenergetic, endocrine and autonomic state	Strong necessity, but its principal age-related failures are mediated through Determinants I, II, III and V rather than constituting a separate hub; see Table 3
Cellular senescence	Terminal state following loss of bioenergetic and structural viability	Downstream of the axis in this architecture; senolytic tractability is emerging, not established
Proteostasis failure	Constitutes Determinant III rather than rivaling the axis	Already inside the architecture as a determinant
Telomere attrition	Accelerated by oxidative load; attenuated by exercise and autonomic regulation	Low modifiability in adulthood by established means
Stem cell exhaustion	Depletion of renewal signaling; niche degradation	Low modifiability in adulthood by established means

**Table 3:** Candidate capacities scored against the three conjunctive criteria. Scores are 0–2 against the anchors stated above.

Candidate Capacity	Independence	Necessity	Modifiability	Outcome	Basis
Bioenergetic capacity	2	2	2	Included (I)	Sets the energetic budget for all active maintenance
Endocrine signaling integrity	2	2	2	Included (II)	Direct mechanistic edge via inner-membrane CYP11A1 step
Molecular quality control	2	2	2	Included (III)	Clearance capacity is not substitutable by energy supply alone
Adaptive (hormetic) stress response	2	2	2	Included (IV)	Responsiveness is separable from, and trainable independent of, throughput
Neuro-autonomic regulation	2	2	2	Included (V)	Sets the state in which the other four operate
Immune competence	1	2	1	Excluded	Principal age-related failures—inflammaging, thymic involution, impaired clearance—are modeled as outputs of I, II, III and V rather than as a separate regulatory capacity
Vascular reserve	1	2	2	Excluded	Endothelial and perfusion decline track bioenergetic and metabolic status; treated as a mediated output of I
Extracellular matrix integrity	2	1	0	Excluded	Genuinely independent, but crosslinking and glycation are not meaningfully reversible in adulthood by established means
Microbiome stability	1	1	2	Excluded	Highly modifiable, but effects on the axis are mediated through II and III
Telomere maintenance	2	1	0	Excluded	Fails modifiability
Stem cell/regenerative capacity	1	2	1	Excluded	Renewal signaling is largely downstream of II; direct modification remains experimental
Musculoskeletal structural integrity	1	2	2	Excluded	Modeled as actuator output of I rather than a regulatory capacity
Cognitive/neural reserve	—	—	—	Excluded by category	An output of the architecture, not a determinant; appears in the biomarker table as a proxy for reserve

### Relation to mTOR Signaling

Any framework proposing a central axis in aging biology must state its relation to mTOR, which integrates nutrient and growth factor sensing with metabolism, autophagy, proteostasis, and mitochondrial biology, and whose inhibition extends lifespan across model organisms [16,17]. We treat mTOR not as a rival hub but as the principal upstream sensor and gate on the axis, with three edges into the architecture.

First, mTORC1 suppresses autophagy through ULK1 phosphorylation [18]; relief of that suppression is the mechanism by which fasting, energy restriction, and sustained exercise raise clearance capacity. mTOR therefore acts on Determinant III, and the compounds and behaviors in the intervention layer that target quality control act substantially through it.

Second, mTORC1 supports mitochondrial biogenesis and oxidative capacity [19], placing it directly on Determinant I. This produces the well-recognized tension in the field: sustained mTORC1 activity favors biogenesis while chronic activity suppresses the clearance that maintains organelle quality. In the language of this framework, that is not a paradox but a control problem—the regulated variable is the ratio of production to clearance, and it is the loss of appropriate cycling between these states, rather than either state alone, that degrades the axis.

Third, mTORC1 couples amino acid availability to insulin expression and β-cell function [20,21], an edge into Determinant II that is often described in metabolic rather than gerontologic terms.

Why, then, is mTOR not the organizing hub? On connectivity, it is a serious candidate. On the second criterion stated in Section 4—therapeutic tractability—it is not, because chronic mTOR inhibition is not an established intervention in otherwise healthy adults, and the interventions that do modulate mTOR in practice are the same behavioral inputs already mapped onto Determinants I, III and IV. Selecting mTOR as the hub would relabel the architecture without changing a single clinical action it recommends. That is a defensible alternative formulation, and we state it as such rather than dismissing it.

## 5. Layer Zero: Biological Starting Conditions

Every individual begins with a unique substrate shaped by genetics, developmental epigenetic programming, and early-life exposures. Layer Zero is not classified as an operational determinant because it is not meaningfully modifiable in adulthood. Its inclusion is explanatory: it accounts for inter-individual heterogeneity in outcomes even when the determinants are implemented with high fidelity. The framework therefore seeks to optimize functional trajectory, not to predict or guarantee absolute lifespan. Importantly—and as a discipline on the model’s falsifiability (Section 11)—Layer Zero is not to be invoked post hoc to rescue the framework from disconfirming cases.

## 6. Framework Methodology—And an Honest Note on Lineage

The framework uses a reductionist systems methodology: organize the biology around a single central axis, then identify the minimum set of distinct, non-substitutable, modifiable capacities required to protect it. Candidate capacities were evaluated against three operational criteria:


**Criterion**

**Test Applied**
IndependenceExerts a primary regulatory influence not fully substitutable by another capacity.NecessityPersistent, uncompensated dysfunction reliably predicts degradation of the central axis.ModifiabilityMeaningfully alterable through behavioral, nutritional, pharmacological, or technological means.

On the methodological lineage—and why this is not a “magic number” argument. This work draws methodological inspiration from Verne Inman’s reduction of human gait to a small set of energy-minimizing mechanisms [22,23]: the philosophy that apparent biological complexity can be organized around a minimum set implementing a single principle. We adopt the philosophy but explicitly do not inherit the number. Inman’s count derives from the mechanics of bipedal locomotion and has itself been substantially revised by later dynamic-walking analyses [24], which showed several classical “determinants” contribute far less than originally claimed. The number of determinants in this framework is therefore a consequence of how many distinct, non-substitutable capacities survive the three criteria above—not a figure carried over from biomechanics. The architecture is also stated so as to be derivable without reference to any specific compound or modality; it is biology-first, not regimen-first.

Evidence grading. STRONG = multiple high-quality RCTs or large prospective studies; MODERATE = consistent observational and interventional data; EMERGING = promising mechanistic and early human data; and EXPERIMENTAL = strong preclinical with limited human data.

### The Decision Matrix

Scoring procedure. Each candidate capacity was scored 0–2 against the three criteria using fixed anchors. Evidence strength is deliberately not one of the criteria; it is reported separately in Section 7, because a capacity can be necessary and modifiable while the human trial base remains thin.

Independence—0: Fully substitutable by, or subsumed within, another candidate. 1: Partially separable, but its principal age-related failures are mediated through other candidates. 2: Exerts a primary regulatory influence not substitutable by another capacity.

Necessity—0: Dysfunction is compensable indefinitely without axis degradation. 1: Dysfunction degrades the axis only in combination with other failures. 2: Persistent, uncompensated dysfunction reliably predicts axis degradation.

Modifiability—0: Not meaningfully alterable in adulthood. 1: Alterable only experimentally or indirectly. 2: Alterable by established behavioral, nutritional, pharmacological, or device-based means.

Inclusion rule. The three criteria are conjunctive gates, not a weighted sum. A capacity is admitted only if it scores 2 on independence and ≥1 on both necessity and modifiability. Because the criteria are conjunctive, no weighting scheme is defined over them, and none can alter the result—a point we return to under sensitivity below.

Sensitivity of the architecture to the thresholds. Because the criteria are conjunctive gates, no weighting scheme is defined over them and none can change the outcome. What does change the outcome is where the thresholds are set, and we state this explicitly rather than defend the number five as a natural constant.

Loosening modifiability to 0—admitting capacities alterable only experimentally—admits extracellular matrix integrity and stem cell regenerative capacity, yielding a seven-capacity architecture. Tightening independence to require no mediated overlap of any kind collapses neuro-autonomic regulation into bioenergetic capacity, since autonomic state acts substantially through energetic and endocrine channels, yielding a four-capacity architecture.

Five is therefore the count that survives the thresholds as stated, not a number carried in from elsewhere. The adjacent architectures are named here so that a reader who disagrees with a threshold can see precisely what the disagreement costs. Advances that make matrix crosslinking or stem cell renewal reliably modifiable in adulthood would move this framework to seven capacities, and we would regard that as the framework working rather than failing.

## 7. The Five Determinants of Functional Reserve

Each determinant is a physiological capacity—a control system whose preservation defends the central axis—not an intervention. The compounds and modalities that act on these capacities are cataloged separately in Section 8.

I. Bioenergetic Capacity—Evidence: STRONG

The foundational substrate·systemic energy throughput and metabolic flexibility

Governs biological energy throughput and thermodynamic efficiency through three coupled components. Circadian entrainment coordinates NAD+ salvage and glymphatic clearance [25,26,27,28]. Physical movement is the primary mechanical stimulus for mitochondrial biogenesis via PGC-1α and among the strongest modifiable predictors of VO2 max and all-cause mortality [9,29,30,31]. Substrate handling determines electron-transport-chain efficiency and metabolic flexibility [32,33]. Within this architecture, Determinant I functions as the gatekeeper: it is modeled as setting the energetic budget available to every other determinant.

- Circadian rhythmicity: NAD+ salvage; glymphatic clearance [25,26];
- Contractile/movement capacity: PGC-1α → mitochondrial biogenesis [9,29];
- Metabolic flexibility: ETC efficiency; substrate switching [32].

II. Endocrine Signaling Integrity—Evidence: MODERATE–STRONG

The macro-systemic communication layer

The communication layer translating environmental and substrate states into coordinated cellular programs—thyroid hormones, sex steroids, GH/IGF-1, and insulin. A direct mechanistic link to the axis distinguishes this capacity: the rate-limiting step of steroidogenesis, cholesterol side-chain cleavage by CYP11A1, occurs on the inner mitochondrial membrane. Because the rate-limiting step is mitochondrial, mitochondrial decline is expected to contribute causally to hormonal decline and not merely to accompany it—though the quantitative contribution in humans has not been established. This capacity cannot be reduced to lifestyle optimization alone.

- Thyroid hormones: Regulation of mitochondrial respiratory rate;
- Sex steroids: Inner-mitochondrial membrane synthesis (CYP11A1);
- GH/IGF-1; insulin: Tissue renewal; substrate selection and allocation.

III. Molecular Quality Control—Evidence: MODERATE

The capacity to clear, refold, and replace damaged components

Reframed from “molecular maintenance” to the underlying capacity: proteostasis, autophagy, mitophagy, and redox buffering—the cell’s ability to remove or repair damaged macromolecules and organelles before they accumulate. This is the determinant that targeted compounds act upon; the compound is a deposit into the capacity, never the capacity itself. It maps directly to the loss-of-proteostasis hallmark.

- Proteostasis and autophagy: Clearance/refolding of damaged proteins;
- Mitophagy: Removal of dysfunctional mitochondria;
- Redox and membrane integrity: Control of electron leakage and lipid peroxidation.

IV. Adaptive (Hormetic) Stress Response—Evidence: EMERGING

The capacity to convert sublethal stress into durable resilience

Reframed from “environmental energy inputs.” The determinant is not the sauna or the cold plunge—those are stimuli. The determinant is the organism’s responsiveness: the capacity to mount and resolve adaptive programs to sublethal thermal, hypoxic, oxidative, and energetic stress (heat shock protein induction, PGC-1α-mediated biogenesis, brown-adipose/UCP1 activation, mitohormesis). This responsiveness declines with age and is itself trainable. Thermal stress shows dose-dependent reductions in cardiovascular mortality in prospective cohorts [34]; photobiomodulation excites cytochrome c oxidase (Complex IV) [35].

- Thermal hormesis: HSP induction; PGC-1α biogenesis [34];
- Hypoxic/oxidative hormesis: Mitohormetic adaptation;
- Photonic input: Complex IV excitation via photobiomodulation [35].

V. Neuro-Autonomic Regulation—Evidence: MODERATE

The integrating condition · the state in which the other four operate

The integrated neurobiological state defined by autonomic balance (high HRV, vagal tone), regulated HPA-axis activity, and low chronic cortisol burden. Mitochondria express high densities of glucocorticoid receptors; chronic sympathetic and cortisol load fragments mitochondrial morphology and suppresses ATP output [36,37]. Conversely, sustained regulation protects telomere length and lowers systemic inflammation [38]. We predict that this capacity modulates the ceiling of the other four; the prediction is specified quantitatively as P4 in Section 11.

- Autonomic balance: HRV; vagal tone;
- HPA regulation: Low chronic cortisol burden [36,37,39];
- Sleep and psychosocial equilibrium: Glymphatic repair [25,26]; purpose, connection.

## 8. The Intervention Layer

**Figure 1:**
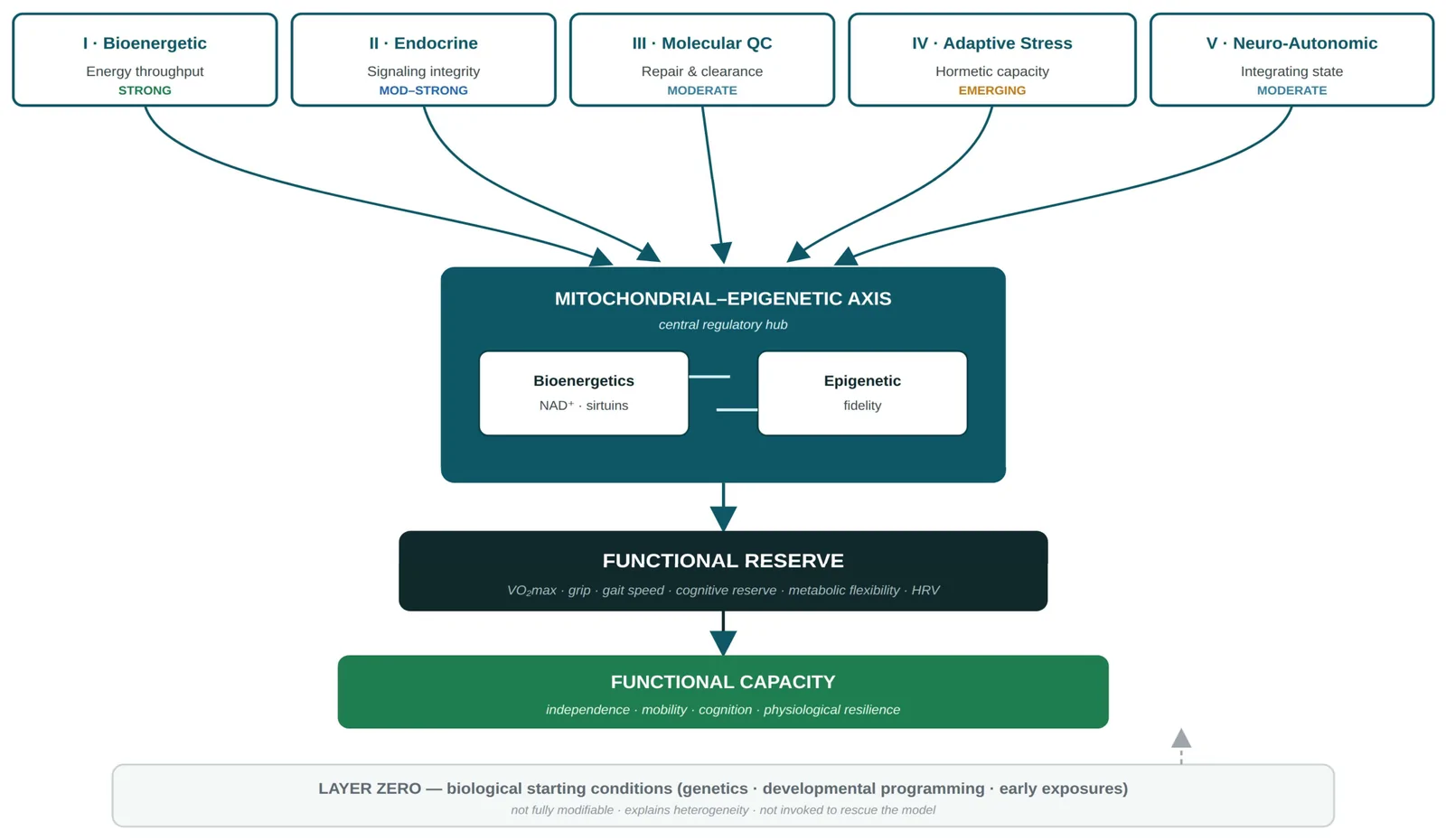
Architecture of the framework. Five modifiable physiological capacities (top) converge on and protect the mitochondrial–epigenetic axis (center), whose stability determines functional reserve and, in turn, functional capacity—the primary clinical outcome. Layer Zero is the non-modifiable substrate that conditions the trajectory. Modifiable interventions (Table 4) act on the determinants, not directly on the outcome. The *number* of capacities shown follows from the inclusion thresholds specified in Section The Decision Matrix rather than from a fixed feature of the biology; Table 3 reports the excluded candidates and the threshold changes that would instead yield four or seven. Arrows indicate direction of protective influence. Colour distinguishes the determinant layer, the central axis, functional reserve, functional capacity, and Layer Zero.

The framework separates two layers that the clinician must not conflate: determinants (the capacities that must be preserved) and interventions (the modifiable inputs that act on them). A useful analogy: functional reserve is a bank balance; interventions are deposits. Exercise, sauna, and a supplement are deposits—they are not the account. Table 4 maps interventions onto their target determinants with evidence grades, and is the appropriate home for compound- and modality-level claims. The architecture is shown in Figure 1.

## 9. Integration with the Hallmarks of Aging

The framework integrates rather than supplants established models [1,2]. The hallmarks are reinterpreted as downstream manifestations of axis instability and capacity failure (Table 5).

## 10. Determinant → Biomarker → Functional Outcome

On the interpretation of these markers. The tiers above are not a ranking of biological importance but of readiness for use in an individual patient, and they behave differently on the four properties that matter for longitudinal assessment.

Sensitivity and specificity. None of these markers is specific to the determinant it indexes. hs-CRP rises with infection, adiposity, and injury as readily as with failure of quality control; HRV falls with acute illness, alcohol, and poor sleep as readily as with autonomic dysregulation. The framework therefore uses them as capacity indices under stable conditions, never as diagnostic tests, and the cross-cutting markers—gait speed, grip strength—are more specific to reserve as a whole than any single-determinant marker is to its determinant.

Reproducibility. This is the property that most sharply separates the tiers, and it constrains what the framework can claim. Gait speed and grip strength have published measurement error, which permits a change in an individual to be interpreted against the known precision of the instrument. VO2 max and HRV are reproducible under standardized conditions and unreliable outside them. The exploratory markers have no established individual-level test–retest performance and cannot at present support serial interpretation in one patient. DunedinPACE warrants specific mention, because Prediction P2 rests on it. Its test–retest reliability is high—the developing authors report an intraclass correlation of 0.96–0.97, achieved in part by restricting the algorithm to probes pre-selected for reliability [45]. High reliability does not, however, imply a small minimum detectable change in an individual. Taking SEM = SD√(1 − ICC) and MDC95 = 2.77 × SEM, an ICC of 0.96 against a cohort standard deviation of 0.10 yields an individual-level MDC95 of approximately 0.055 pace units—large relative to the effect sizes reported in intervention studies. P2 is accordingly specified in Section 11 as a cohort-level rather than an individual-level test, where the standard error of a group mean scales with sample size. We note also that some published test–retest evaluations were conducted in the dataset from which probe reliability was itself estimated, and we therefore treat the reported figures as upper bounds.

Feasibility for longitudinal assessment. The validated tier is measurable in ordinary practice at intervals of six to twelve months at acceptable cost and burden. The candidate tier is measurable but not yet standardized enough for the results to be compared across sites or instruments. The exploratory tier is not feasible outside a research setting and is listed to identify where instrument development is required—which is the substance of Limitation 5, not a claim of present clinical utility.

## 11. Falsifiability and Testable Predictions

The framework is offered as a scientific hypothesis and must be falsifiable in practice, not merely in principle. We acknowledge the standard failure mode of grand frameworks: a model with an escape hatch for every observation can never be disconfirmed. To avoid this, we pre-commit to specific risky predictions and constrain the escape hatches (notably, Layer Zero is not available as a post hoc rescue; Section 5).

Each prediction below specifies an effect size threshold, an observation window, a minimum covariate set, and separate criteria for support and for falsification. Thresholds are anchored to published measurement error where it exists, so that a null result can be distinguished from a result the instrument could not have detected. Where a threshold is set by convention rather than by measurement error, we say so (Table 6).

**Table 6:** Testable predictions, quantified. Each carries an effect size threshold, an observation window, a minimum covariate set, and separate criteria for support and for falsification. P1 is the primary test and interrogates the number of determinants directly. All six are evaluable against existing longitudinal cohorts and registries; none requires new data collection.

No.	Prediction and Specification
P1 (primary)	Removal of any one determinant from a model of longitudinal functional reserve trajectory produces a statistically significant decrement in explanatory power on nested model comparison—the deletion test. Threshold: Removal of any one determinant from a model of longitudinal functional reserve trajectory produces a statistically significant decrement in explanatory power on nested model comparison, and that decrement is not recoverable by refitting weights on the remaining four. Window: Existing cohorts with three or more waves—Health ABC, BLSA. No new data collection. Covariates: Age, sex, race, baseline reserve proxy, comorbidity count. Support: Every one of the five removals degrades model fit. Falsification: If removing a determinant does not degrade fit, or if reweighting the remaining four fully recovers it, that determinant is not independently necessary and the architecture is over-specified. This is a direct, quantitative test of the number five.
P2	Multi-determinant optimization produces DunedinPACE deceleration whose effect size increases with the number of determinants concurrently optimized. Threshold: The 3+ determinant stratum must differ from the 1-determinant stratum by a standardized effect of at least 0.25 SD in DunedinPACE, benchmarked to the 24-month standardized treatment effect of 25% caloric restriction in the CALERIE trial: d = −0.25 (95% CI −0.41, −0.09), *p* < 0.003 [46]. The 12-month effect was d = −0.29 (95% CI −0.45, −0.13); the 24-month figure is used because P2 specifies a 24-month window. Specifying the threshold in standardized units removes any dependence on a chosen cohort standard deviation—which matters, because CALERIE’s baseline DunedinPACE mean and SD appear only in the supplementary material of that source publication. Power anchor. CALERIE detected this effect with 117 treatment and 68 control participants contributing baseline-to-24-month change (197 total contributed baseline plus at least one follow-up). Any test of P2 should be powered against those numbers, not against the headline randomized n of 220. Why this threshold, and why cohort-level. It is set at, not below, the largest effect demonstrated in a randomized trial of a single multi-system intervention: a multi-determinant architecture that cannot match a single well-executed one does not warrant its added complexity. CALERIE’s effect corresponded to roughly 3% slowing, on the order of 0.03 pace units. An illustrative individual-level MDC95, derived from ICC 0.96 and an assumed cohort SD of 0.10, is approximately 0.055—close to twice that. This figure is illustrative only and is not load-bearing: because the threshold is stated in standardized units, no claim depends on the assumed SD. The strongest human caloric restriction RCT therefore produced an effect about half the size of what is detectable in a single person, which is the concrete reason P2 is specified as a cohort-level test. Window: Minimum 24 months; DunedinPACE indexes rate, and shorter windows cannot resolve a rate change from assay noise. Covariates: Age, sex, baseline pace, BMI, smoking, comorbidity count, socioeconomic index. Support: Monotonic increase across strata, adjusted, with confidence intervals excluding the null in the 3+ stratum. Falsification: Absence of dose–response. A non-monotonic result attributable to intervention interference does not falsify, as it bears on additivity rather than architecture—this exception is declared in advance and no further exceptions will be admitted post hoc. Level: Cohort, not individual. See the reproducibility note under Table 7.
P3	Interventions on Determinants III–V show attenuated efficacy under severe Determinant I deficiency. Threshold: An interaction term for baseline bioenergetic stratum reaching at least a 30% relative attenuation of intervention effect between the lowest and highest baseline VO2 max tertiles. Window: 12 months minimum. Covariates: Age, sex, baseline value of the targeted determinant, adherence, comorbidity count. Support: Significant interaction in the predicted direction. Falsification: Equivalent efficacy across bioenergetic strata, adequately powered, falsifies the contingent hierarchy. Existing evidence. The closest existing evidence is a secondary analysis of the Testosterone Trials. The Physical Function Trial did not meet its primary endpoint, but across participants, the treatment effect on self-reported physical function was significantly greater in men whose baseline walking speed was at or above 1.2 m/s (4.9, 95% CI 2.2–7.7) than below it (2.5, 95% CI 0.29–4.6), with a significant interaction—a result the investigators noted ran contrary to their prior expectation [47]. An endocrine intervention producing more benefit where function is more intact is consistent with the contingent hierarchy. It is not a test of it: baseline gait speed is a cross-cutting reserve proxy rather than a measure of bioenergetic capacity, and the attenuation may equally reflect competing pathology in slower walkers that testosterone cannot address. We cite it to show the prediction is not idle, not to claim it is confirmed.
P4	Neuro-autonomic dysregulation predicts reduced trainability across the other determinants. Threshold: Individuals with chronic low HRV or elevated morning cortisol show attenuated trainability—ΔVO2 max or Δstrength per unit training load—relative to matched controls with intact autonomic markers, independent of baseline fitness; demonstrable as a significant interaction between baseline HRV tertile and intervention response. Window: 12 months. Testable in existing cardiac rehabilitation datasets. Covariates: Age, sex, baseline value of targeted determinant, medications affecting autonomic tone (beta-blockers, anticholinergics), sleep duration. Support: Significant interaction, lowest HRV tertile showing the smallest response. Falsification: No ceiling effect in an adequately powered analysis falsifies Determinant V’s modulating role.
P5	No cohort sustains exceptional multi-systemic function with chronic uncompensated deficiency in a single determinant’s core mechanism. Threshold: One documented counterexample with objective multi-systemic function in the top decile for age and documented persistent deficiency, not a transient or compensated one. Window: Cross-sectional against existing registries. Covariates: Not applicable; this is an existence claim. Support: Absence of counterexamples across centenarian and Blue Zone registries. Falsification: One robust counterexample falsifies that determinant’s necessity, decisively, with no Layer Zero appeal.
P6	Rate of change in a reserve proxy predicts functional outcome beyond its level. Threshold: Annualized gait speed slope adds significant predictive information over baseline gait speed in a joint model. Window: ≥4 years with at least three measurements. Covariates: Age, sex, race, baseline speed, comorbidity count. Support: Slope retains significance with level in the model. Falsification: Slope adds nothing over level. Scope. The full treatment of point-versus-trajectory measurement is developed separately and is under review elsewhere; P6 states only the prediction this framework requires.

**Table 7:** Each determinant linked to validated or candidate biomarkers and the functional output it governs. Cross-cutting markers index reserve as a whole. Tier definitions: VALIDATED—clinically available, established reference ranges, prospective outcome data. CANDIDATE—clinically measurable and outcome-associated, but not standardized for this use. EXPLORATORY—research-grade assay only.

Determinant	Candidate Biomarker	Tier	Functional Outcome
I Bioenergetic	VO2 max [9]	VALIDATED	Aerobic capacity; metabolic resilience
	Lactate threshold	CANDIDATE	
	CGM-derived glucose variability	CANDIDATE	
II Endocrine	Thyroid panel	VALIDATED	Anabolic capacity; body composition
	Free testosterone/estradiol	VALIDATED	
	IGF-1	CANDIDATE	
	Fasting insulin (HOMA-IR)	VALIDATED	
III Molecular QC	hs-CRP	VALIDATED	Tissue integrity; damage clearance rate
	Oxidized LDL	EXPLORATORY	
	Autophagy markers	EXPLORATORY	
IV Adaptive stress	VO2 trainability	CANDIDATE	Resilience to acute stressors
	HSP induction	EXPLORATORY	
	Cold/heat tolerance	EXPLORATORY	
V Neuro-autonomic	HRV (RMSSD)	VALIDATED	Recovery; cognitive and affective stability
	Morning cortisol/DHEA	CANDIDATE	
	Sleep architecture	VALIDATED by polysomnography; CANDIDATE by wearable	
Cross-cutting	Gait speed [10]	VALIDATED	Integrated functional reserve
	Grip strength	VALIDATED	
	DunedinPACE [45]	CANDIDATE	

## 12. Limitations

Intellectual integrity requires explicit acknowledgment of the framework’s limitations.

1 Synthetically derived, not empirically derived. The determinants were identified through clinical reasoning and literature synthesis, not from a primary prospective dataset. This is a structured hypothesis, not a validated theory; prospective validation must precede any clinical guideline.

2 Heterogeneous evidence base. Evidence ranges from strong (Determinant I) to emerging (Determinant IV). The necessity scoring in Section The Decision Matrix is a claim about the architecture’s internal logic and is deliberately separate from the strength of the human evidence supporting each capacity: a capacity may be structurally necessary while its trial base remains thin. Determinant IV carries the weakest evidence of the five, and its necessity claim is accordingly the most exposed of the five. Prediction P5 applied to the adaptive stress response is the test most likely to disconfirm this framework, and we identify it as such rather than leaving it to be found.

3 The organizing principle is proposed, not proven. While the NAD+/sirtuin/mitochondrial cascade is well documented and its epigenetic arm is at least partially reversible [11,12,48], the claim that the mitochondrial–epigenetic axis is the primary organizing principle—rather than telomere attrition, proteostasis failure, or inflammaging—remains contested and cannot be excluded on current evidence.

4 Overlap with established constructs. Functional reserve, homeostenosis, physiologic resilience, and frailty are established in geriatric medicine [4,5]. Physiological complexity, in the Lipsitz and Goldberger tradition, is a further adjacent construct that this framework reorganizes rather than originates; Section 2 states the distinction. This framework reorganizes and operationalizes these constructs around a mechanistic axis; it does not originate them, and should be read as integrative rather than wholly novel.

5 No companion measurement protocol. The framework specifies what must be preserved but not yet a standardized, cross-validated instrument for each determinant. Table 7 lists candidates; validated assessment protocols remain to be developed.

6 *n* = 1 derivation and stack independence. Elements were informed by the author’s monitored self-experimentation, which provides clinical face validity but not population-level evidence. To guard against rationalizing a personal regimen, the determinant architecture is stated so as to be derivable without reference to any specific compound; all compound-level claims are confined to the intervention layer and carry its lower evidence weight.

Use of AI tools: Large language models were used for copy editing and manuscript preparation. All scientific ideas, claims, and the framework are the author’s own, and the author takes full responsibility for the content.

Preprint: An earlier version of this manuscript has been deposited on Preprints.org (https://doi.org/10.20944/preprints202607.1589.v1).

## 13. Discussion and Clinical Implications

The primary contribution is architectural, not mechanistic—the individual mechanistic claims are inherited from the established literature. The contribution is the proposal that aging biology can be organized around a single bidirectional axis and a minimum set of modifiable capacities whose preservation maintains functional reserve, and that this organization has direct clinical utility. The control systems reframe shifts the clinical question from “what goes wrong?” to “what must go right?”

The contingent hierarchy. Each determinant depends on the integrity of those preceding it. Receptor-mediated and molecular interventions are degraded in an insulin-resistant, circadian-disrupted cellular environment because the bioenergetic substrate required to execute them is absent. The hierarchy is not absolute—partial benefit from higher determinants can occur with incomplete lower-determinant optimization—but persistent uncompensated failure in a lower determinant is predicted to cap the ceiling of all higher ones.

On “self-reference”—stated modestly. Mitochondria contribute to several determinants: they fund the energetic budget (I), perform the inner-membrane steroidogenic step (II), and encode stress-signaling peptides (Table 4). We note this convergence as consistent with the axis’s centrality, but we explicitly decline to elevate it to a deep structural theorem. Mitochondrial multifunctionality is expected, not surprising, and we do not rest any load-bearing claim on it.

Therapeutic prioritization. The contingent hierarchy implies that Determinant I should yield the highest expected marginal return across subsequent determinants. This is a consequence of the architecture, not an evidence-based recommendation, and it is stated here as a hypothesis that P3 tests. If the hierarchy holds, initial clinical evaluation would be expected to address circadian health, physical activity capacity, and metabolic flexibility before higher-determinant assessment or the costlier options in the intervention layer. We do not offer this as a practice recommendation; the hierarchy is untested.

What the framework offers at the point of care now. Three things, none of which requires the framework itself to be validated first.

A sequence for assessment. The determinants are ordered, and the ordering indicates what to establish before spending a patient’s time or money further up. In a patient whose sleep is fragmented, whose activity capacity is low, and whose substrate handling is impaired, higher-determinant assessment and the costlier options in the intervention layer are predicted to underperform. This is a triage heuristic rather than a guideline, and P3 tests it directly.

A distinction the clinical conversation usually lacks—between a capacity and a deposit into it. Patients arrive asking about compounds. The two-layer structure lets a clinician answer that question without either dismissing it or endorsing it: the compound is evaluated by which capacity it serves and what evidence grade it carries, and the capacity remains the target whether or not that particular compound survives scrutiny.

A measurement discipline. The biomarker table separates markers usable in an individual patient today from those that are not, and states the interval at which the validated tier can meaningfully be repeated. Gait speed, grip strength, VO2 max, heart rate variability, and standard metabolic and endocrine panels can be acted on now, because each carries predictive validity independent of this framework.

On magnitude. The effects available here are small per unit time and consequential in aggregate. In prospective cohorts, each standard deviation increment in DunedinPACE carries a mortality hazard ratio of 1.26 (95% CI 1.14–1.40) in the Normative Aging Study and 1.65 (95% CI 1.51–1.79) in the Framingham Heart Study Offspring cohort [45]. A quarter-standard-deviation difference—the magnitude produced by two years of 25% caloric restriction in the CALERIE trial [46]—corresponds on those associations to roughly 6–12% lower mortality hazard. These are observational associations rather than demonstrated effects of intervention, and the conversion assumes the association is approximately log-linear across this range. We state them to indicate the scale of what is at stake, not to promise it. That scale is why the framework treats reserve, rather than lifespan, as the quantity to be measured and defended.

Research design. The falsifiability criteria generate hypotheses directly evaluable against existing longitudinal datasets—centenarian registries, Blue Zone cohorts, super-ager studies—without new data collection. The framework also inherits the predictive validity of its named outputs [9,10,45].

## 14. Conclusions

We have proposed a unifying, systems-oriented hypothesis that reframes human functional aging as progressive instability within a coupled, bidirectional mitochondrial–epigenetic axis, and proposes five modifiable physiological capacities required to preserve functional reserve. A separate intervention layer maps modifiable inputs onto those capacities, keeping the distinction between what must be preserved and what is done to preserve it.

Functional reserve is advanced as the primary therapeutic target; lifespan is its downstream consequence. The framework’s distinguishing features—the control systems inversion, the explicit reserve construct anchored in existing geriatric science, the two-layer determinant/intervention separation, and an honestly constrained falsifiability—differentiate it from purely descriptive models. It is offered not as settled science but as a structured hypothesis that meets the first requirement of productive scientific discourse: it can be proven wrong.

## Figures and Tables

**Table 1:** Aging reframed as failure modes of a biological control system.

Control Failure	Physiological Correlate	Functional Consequence
Loss of gain	Blunted corrective responses (attenuated trainability; weakened heat shock and antioxidant induction)	Slower, smaller recovery from any perturbation
Sensor corruption	Deregulated nutrient sensing; insulin/leptin resistance	Fuel misallocation; metabolic inflexibility
Actuator failure	Sarcopenia; reduced mitochondrial density and output	Diminished force, power, and energetic ceiling
Setpoint drift	Epigenetic drift; loss of transcriptional fidelity [3]	Progressive identity loss in differentiated cells

**Table 4:** Modifiable interventions mapped onto the five determinants. Compound- and modality-level claims carry the evidence weight shown here, not the structural status of the determinants themselves. Rows are ordered by strength of human evidence, not by expected effect size or by position in the architecture. The final row is separated to mark that it is offered as a future direction and not as present recommendation. The experimental intervention rows are deliberately excluded from the necessity claim.

Intervention	Target	Principal Mechanism	Evidence
Aerobic + resistance exercise	I (+III, IV)	PGC-1α biogenesis; mitophagy; hormetic adaptation	STRONG
Circadian & sleep hygiene	I (+V)	NAD+ salvage; glymphatic clearance	STRONG
Dietary pattern/energy balance	I (+II)	ETC efficiency; metabolic flexibility; nutrient sensing	STRONG–MOD
NAD+ precursors (NMN/NR)	III	Sustain sirtuin substrate availability [11,12,40]	MODERATE
CoQ10·omega-3·creatine	III	Electron leak control; membrane fluidity; energy buffering	MODERATE
HRV/contemplative training·psychosocial purpose	V	Vagal tone; HPA regulation; reduced inflammatory load [38,41]	MODERATE
Hormone optimization (where indicated)	II	Restoration of deficient endocrine signaling	MOD (context)
Urolithin A	III	Mitophagy activation; clearance of damaged mitochondria	EMERGING
Sauna·cold·photobiomodulation·hypoxic conditioning·time-restricted eating	IV	Hormetic induction (HSP, UCP1, mitohormesis, Complex IV) [34,35,42]	EMERGING
Mitochondrial-derived & targeted peptides (MOTS-c, secretagogues, bioregulators)	III/V signaling	Receptor-mediated transcriptional programs; mtDNA-encoded stress signaling [43,44]	EXPERIMENTAL

**Table 5:** Mapping of the hallmarks of aging [1,2] to the framework’s determinants and central axis.

Hallmark of Aging	Primary Mapping	Systems Interpretation
Mitochondrial dysfunction	Central axis + all determinants	Degradation of the master energetic engine
Epigenetic alterations	Central axis	Information loss within the primary control system [3]
Deregulated nutrient sensing	Determinant I + II	Sensor corruption; fuel misallocation
Loss of proteostasis	Determinant III	Failure of molecular quality control capacity
Cellular senescence	Determinants I–V (systemic)	Terminal arrest after loss of bioenergetic/structural viability
Stem cell exhaustion	Determinant II (+ signaling)	Depletion of renewal signals; niche degradation
Chronic inflammation	Determinants I–V (network)	Burden from accumulated uncompensated damage
Altered intercellular comm.	Determinant II + V	Corrupted endocrine and neuroendocrine transmission
Genomic instability	Layer Zero/Determinant III	Primary substrate; partly addressable via quality control
Telomere attrition	Determinant I + V (indirect)	Accelerated by oxidative stress; attenuated by exercise [29] and regulation [38]

## Data Availability

No new data were created or analyzed in this study. Data sharing is not applicable to this article.
